# Diaqua­bis(2-chloro­benzoato-κ*O*)bis­(*N*,*N*-diethyl­nicotinamide-κ*N* ^1^)nickel(II)

**DOI:** 10.1107/S1600536809014226

**Published:** 2009-04-22

**Authors:** Tuncer Hökelek, Hakan Dal, Barış Tercan, F. Elif Özbek, Hacali Necefoğlu

**Affiliations:** a Department of Physics, Hacettepe University, 06800 Beytepe, Ankara, Turkey; b Department of Chemistry, Faculty of Science, Anadolu University, 26470 Yenibağlar, Eskişehir, Turkey; c Department of Physics, Karabük University, 78050 Karabük, Turkey; d Department of Chemistry, Kafkas University, 63100 Kars, Turkey

## Abstract

In the monomeric and centrosymmetric title Ni^II^ complex, [Ni(C_7_H_4_ClO_2_)_2_(C_10_H_14_N_2_O)_2_(H_2_O)_2_], the Ni^II^ ion is located on an inversion center. The asymmetric unit contains one 2-chloro­benzoate ligand, one diethyl­nicotinamide (DENA) ligand and one coordinating water mol­ecule, the ligands being monodentate. The four O atoms in the equatorial plane around the Ni^II^ ion form a slightly distorted square-planar arrangement, while the slightly distorted octa­hedral coordination is completed by two N atoms of the DENA ligands in axial positions. The dihedral angle between the benzene ring and the attached carboxyl­ate group is 87.36 (10)°, while the pyridine and benzene rings are oriented at an angle of 41.90 (5)°. In the crystal structure, inter­molecular O—H⋯O hydrogen bonds link the mol­ecules into a two-dimensional network parallel to (10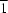).

## Related literature

For general backgroud, see: Antolini *et al.* (1982 ▶); Bigoli *et al.* (1972 ▶); Nadzhafov *et al.* (1981 ▶); Shnulin *et al.* (1981 ▶). For related structures, see: Hökelek *et al.* (1995 ▶, 1997 ▶, 2007 ▶, 2008 ▶); Hökelek & Necefoğlu (1996 ▶, 1997 ▶, 2007 ▶).
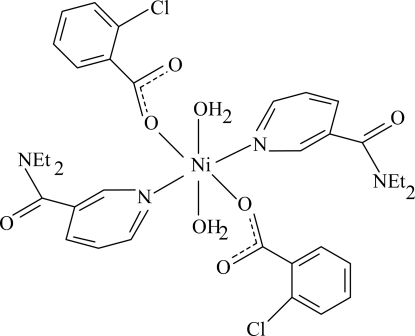


## Experimental

### 

#### Crystal data

[Ni(C_7_H_4_ClO_2_)_2_(C_10_H_14_N_2_O)_2_(H_2_O)_2_]
*M* *_r_* = 762.31
Monoclinic, 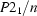

*a* = 12.7505 (2) Å
*b* = 10.3565 (2) Å
*c* = 14.9673 (3) Å
β = 114.046 (1)°
*V* = 1804.92 (6) Å^3^
*Z* = 2
Mo *K*α radiation
μ = 0.74 mm^−1^
*T* = 100 K
0.27 × 0.18 × 0.11 mm

#### Data collection

Bruker Kappa APEXII CCD area-detector diffractometer
Absorption correction: multi-scan (*SADABS*; Bruker, 2005 ▶) *T*_min_ = 0.828, *T*_max_ = 0.923
16593 measured reflections
4519 independent reflections
3781 reflections with *I* > 2σ(*I*)
*R*_int_ = 0.034

#### Refinement

*R*[*F* ^2^ > 2σ(*F* ^2^)] = 0.031
*wR*(*F* ^2^) = 0.084
*S* = 1.06
4519 reflections
233 parameters
2 restraints
H atoms treated by a mixture of independent and constrained refinement
Δρ_max_ = 1.08 e Å^−3^
Δρ_min_ = −0.39 e Å^−3^

### 

Data collection: *APEX2* (Bruker, 2007 ▶); cell refinement: *SAINT* (Bruker, 2007 ▶); data reduction: *SAINT*; program(s) used to solve structure: *SHELXS97* (Sheldrick, 2008 ▶); program(s) used to refine structure: *SHELXL97* (Sheldrick, 2008 ▶); molecular graphics: *ORTEP-3 for Windows* (Farrugia, 1997 ▶); software used to prepare material for publication: *WinGX* (Farrugia, 1999 ▶).

## Supplementary Material

Crystal structure: contains datablocks I, global. DOI: 10.1107/S1600536809014226/ci2776sup1.cif

Structure factors: contains datablocks I. DOI: 10.1107/S1600536809014226/ci2776Isup2.hkl

Additional supplementary materials:  crystallographic information; 3D view; checkCIF report

## Figures and Tables

**Table 1:** Selected bond lengths (Å)

Ni1—O1	2.0336 (10)
Ni1—O4	2.0867 (10)
Ni1—N1	2.1181 (12)

**Table 2:** Hydrogen-bond geometry (Å, °)

*D*—H⋯*A*	*D*—H	H⋯*A*	*D*⋯*A*	*D*—H⋯*A*
O4—H41⋯O2^i^	0.82 (2)	1.86 (2)	2.6267 (17)	155 (2)
O4—H42⋯O3^ii^	0.85 (2)	1.93 (2)	2.7826 (15)	172 (2)

Symmetry codes: (i) 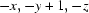; (ii) 
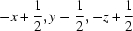
.

**Fig. 1.**
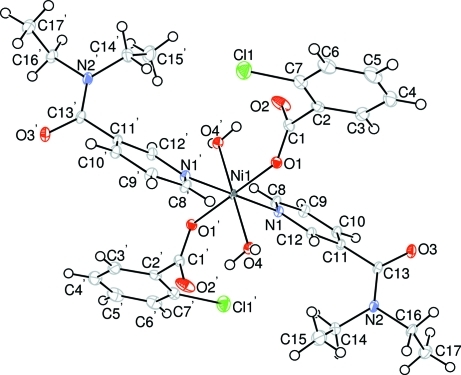
The molecular structure of the title compound with the atom-numbering scheme. Displacement ellipsoids are drawn at the 50% probability level. Primed atoms are generated by the symmetry operator (- x, 1 -y, -z).

## supplementary crystallographic information

### Comment

Transition metal complexes with biochemically active ligands frequently show interesting physical and/or chemical properties, as a result they may find applications in biological systems (Antolini *et al.*, 1982). The structural functions and coordination relationships of the arylcarboxylate ion in transition metal complexes of benzoic acid derivatives change depending on the nature and position of the substituent groups on the benzene ring, the nature of the additional ligand molecule or solvent, and the medium of the synthesis (Nadzhafov *et al.*, 1981; Shnulin *et al.*, 1981). The nicotinic acid derivative *N*,*N*-diethylnicotinamide (DENA) is an important respiratory stimulant (Bigoli *et al.*, 1972).

The structure determination of the title compound, (I), a nickel complex with two 2-chlorobenzoate (CB), two diethylnicotinamide (DENA) ligands and two water molecules, was undertaken in order to determine the properties of the ligands and also to compare the results obtained with those reported previously.

Compound (I) is a monomeric complex, with the Ni^II^ ion on a centre of symmetry. It contains two CB, two DENA ligands and two water molecules (Fig. 1). All ligands are monodentate. The four O atoms (O1, O4, and the symmetry-related atoms O1', O4') in the equatorial plane around the Ni^II^ ion form a slightly distorted square-planar arrangement, while the slightly distorted octahedral coordination is completed by the two N atoms of the DENA ligands (N1, N1') in the axial positions (Table 1 and Fig. 1).

The near equality of the C1—O1 [1.2616 (17) Å] and C1—O2 [1.2435 (18) Å] bonds in the carboxylate group indicates a delocalized bonding arrangement, rather than localized single and double bonds, and may be compared with the corresponding distances: 1.256 (6) and 1.245 (6) Å in [Mn(DENA)_2_(C_7_H_4_ClO_2_)_2_(H_2_O)_2_], (II) (Hökelek *et al.*, 2008), 1.265 (6) and 1.275 (6) Å in [Mn(C_9_H_10_NO_2_)_2_(H_2_O)_4_].2(H_2_O), (III) (Hökelek & Necefoğlu, 2007), 1.260 (4) and 1.252 (4) Å in [Zn(DENA)_2_(C_7_H_4_FO_2_)_2_(H_2_O)_2_],(IV) (Hökelek *et al.*, 2007), 1.259 (9) and 1.273 (9) Å in Cu_2_(DENA)_2_(C_6_H_5_COO)_4_, (V) (Hökelek *et al.*, 1995), 1.279 (4) and 1.246 (4) Å in [Zn_2_(DENA)_2_(C_7_H_5_O_3_)_4_].2H_2_O, (VI) (Hökelek & Necefoğlu, 1996), 1.251 (6) and 1.254 (7) Å in [Co(DENA)_2_(C_7_H_5_O_3_)_2_(H_2_O)_2_], (VII) (Hökelek & Necefoğlu, 1997) and 1.278 (3) and 1.246 (3) Å in [Cu(DENA)_2_(C_7_H_4_NO_4_)_2_(H_2_O)_2_], (VIII) (Hökelek *et al.*, 1997). In (I), the average Ni—O bond length is 2.0602 (10) Å and the Ni1 atom is displaced out of the least-squares plane of the carboxylate group (O1/C1/O2) by -0.276 (1) Å. The dihedral angle between the planar carboxylate group and the benzene ring A (C2—C7) is 87.36 (10)°, while that between rings A and B (N1/C8—C12) is 41.90 (5)°.

In the crystal structure, intermolecular O—H···O hydrogen bonds (Table 2) link the molecules into a two-dimesional network parallel to the (1 0 1).

### Experimental

The title compound was prepared by the reaction of Ni(SO_4_).6(H_2_O) (1.31 g, 5 mmol) in H_2_O (20 ml) and DENA (1.78 g, 10 mmol) in H_2_O (20 ml) with sodium 2-chlorobenzoate (1.785 g, 10 mmol) in H_2_O (50 ml). The mixture was filtered and set aside to crystallize at ambient temperature for 3 d, giving blue single crystals.

### Refinement

H atoms of water molecule were located in a difference Fourier map and refined isotropically, with a O-H restraint. The remaining H atoms were positioned geometrically with C-H = 0.93, 0.97 and 0.96 Å, for aromatic, methylene and methyl H atoms and constrained to ride on their parent atoms, with *U*_iso_(H) = *xU*_eq_(C), where *x* = 1.5 for methyl H and *x* = 1.2 for all other H atoms.

### Crystal data

**Table:**

[Ni(C_7_H_4_ClO_2_)_2_(C_10_H_14_N_2_O)_2_(H_2_O)_2_]	*F*(000) = 796
*M**_r_* = 762.31	*D*_x_ = 1.403 Mg m^−^^3^
Monoclinic, *P*2_1_/*n*	Mo *K*α radiation, λ = 0.71073 Å
Hall symbol: -P 2yn	Cell parameters from 7783 reflections
*a* = 12.7505 (2) Å	θ = 2.5–28.4°
*b* = 10.3565 (2) Å	µ = 0.74 mm^−^^1^
*c* = 14.9673 (3) Å	*T* = 100 K
β = 114.046 (1)°	Block, blue
*V* = 1804.92 (6) Å^3^	0.27 × 0.18 × 0.11 mm
*Z* = 2	

### Data collection

**Table:**

Bruker Kappa APEXII CCD area-detector diffractometer	4519 independent reflections
Radiation source: fine-focus sealed tube	3781 reflections with *I* > 2σ(*I*)
graphite	*R*_int_ = 0.034
φ and ω scans	θ_max_ = 28.4°, θ_min_ = 1.8°
Absorption correction: multi-scan (*SADABS*; Bruker, 2005)	*h* = −17→17
*T*_min_ = 0.828, *T*_max_ = 0.923	*k* = −13→12
16593 measured reflections	*l* = −20→18

### Refinement

**Table:**

Refinement on *F*^2^	Primary atom site location: structure-invariant direct methods
Least-squares matrix: full	Secondary atom site location: difference Fourier map
*R*[*F*^2^ > 2σ(*F*^2^)] = 0.031	Hydrogen site location: inferred from neighbouring sites
*wR*(*F*^2^) = 0.084	H atoms treated by a mixture of independent and constrained refinement
*S* = 1.06	*w* = 1/[σ^2^(*F*_o_^2^) + (0.0456*P*)^2^ + 0.2566*P*] where *P* = (*F*_o_^2^ + 2*F*_c_^2^)/3
4519 reflections	(Δ/σ)_max_ = 0.001
233 parameters	Δρ_max_ = 1.08 e Å^−^^3^
2 restraints	Δρ_min_ = −0.39 e Å^−^^3^

### Special details

**Table:**

Geometry. All e.s.d.'s (except the e.s.d. in the dihedral angle between two l.s. planes) are estimated using the full covariance matrix. The cell e.s.d.'s are taken into account individually in the estimation of e.s.d.'s in distances, angles and torsion angles; correlations between e.s.d.'s in cell parameters are only used when they are defined by crystal symmetry. An approximate (isotropic) treatment of cell e.s.d.'s is used for estimating e.s.d.'s involving l.s. planes.
Refinement. Refinement of *F*^2^ against ALL reflections. The weighted *R*-factor *wR* and goodness of fit *S* are based on *F*^2^, conventional *R*-factors *R* are based on *F*, with *F* set to zero for negative *F*^2^. The threshold expression of *F*^2^ > σ(*F*^2^) is used only for calculating *R*-factors(gt) *etc*. and is not relevant to the choice of reflections for refinement. *R*-factors based on *F*^2^ are statistically about twice as large as those based on *F*, and *R*- factors based on ALL data will be even larger.

### Fractional atomic coordinates and isotropic or equivalent isotropic displacement parameters (Å^2^)

**Table:**

	*x*	*y*	*z*	*U*_iso_*/*U*_eq_	
Ni1	0.0000	0.5000	0.0000	0.00853 (8)	
Cl1	−0.31050 (4)	0.35043 (4)	0.08471 (3)	0.02678 (11)	
O1	−0.03038 (8)	0.44605 (10)	0.11794 (7)	0.0128 (2)	
O2	−0.13227 (11)	0.61462 (11)	0.13322 (9)	0.0268 (3)	
O3	0.43014 (9)	0.61598 (10)	0.39621 (7)	0.0151 (2)	
O4	0.11786 (9)	0.34845 (10)	0.03504 (8)	0.0123 (2)	
H41	0.1299 (19)	0.338 (2)	−0.0143 (13)	0.042 (6)*	
H42	0.1100 (18)	0.2746 (17)	0.0570 (15)	0.043 (6)*	
N1	0.13740 (10)	0.61957 (11)	0.08958 (9)	0.0114 (2)	
N2	0.48379 (11)	0.49545 (12)	0.29580 (9)	0.0152 (3)	
C1	−0.09075 (13)	0.50440 (14)	0.15415 (11)	0.0135 (3)	
C2	−0.11211 (13)	0.42820 (14)	0.23115 (11)	0.0148 (3)	
C3	−0.03393 (14)	0.42920 (16)	0.32862 (12)	0.0202 (3)	
H3	0.0299	0.4830	0.3480	0.024*	
C4	−0.05013 (15)	0.35049 (17)	0.39746 (12)	0.0259 (4)	
H4	0.0027	0.3517	0.4624	0.031*	
C5	−0.14508 (16)	0.27062 (17)	0.36887 (13)	0.0270 (4)	
H5	−0.1551	0.2169	0.4145	0.032*	
C6	−0.22513 (15)	0.27003 (16)	0.27308 (13)	0.0235 (4)	
H6	−0.2895	0.2170	0.2541	0.028*	
C7	−0.20830 (14)	0.34949 (15)	0.20560 (11)	0.0183 (3)	
C8	0.14003 (12)	0.74683 (14)	0.07426 (11)	0.0136 (3)	
H8	0.0804	0.7833	0.0209	0.016*	
C9	0.22780 (12)	0.82654 (14)	0.13450 (11)	0.0151 (3)	
H9	0.2255	0.9148	0.1223	0.018*	
C10	0.31847 (12)	0.77378 (14)	0.21268 (11)	0.0139 (3)	
H10	0.3776	0.8255	0.2547	0.017*	
C11	0.31889 (12)	0.64090 (14)	0.22689 (10)	0.0119 (3)	
C12	0.22679 (12)	0.56819 (14)	0.16507 (10)	0.0124 (3)	
H12	0.2267	0.4799	0.1762	0.015*	
C13	0.41594 (12)	0.58190 (13)	0.31243 (11)	0.0120 (3)	
C14	0.47757 (15)	0.45998 (18)	0.19868 (12)	0.0254 (4)	
H14A	0.5535	0.4670	0.1989	0.031*	
H14B	0.4278	0.5205	0.1507	0.031*	
C15	0.43258 (16)	0.32361 (19)	0.16849 (14)	0.0368 (5)	
H15A	0.4372	0.3023	0.1078	0.055*	
H15B	0.3541	0.3187	0.1605	0.055*	
H15C	0.4781	0.2638	0.2181	0.055*	
C16	0.58189 (13)	0.44280 (15)	0.38015 (11)	0.0166 (3)	
H16A	0.5967	0.3551	0.3657	0.020*	
H16B	0.5628	0.4401	0.4366	0.020*	
C17	0.68933 (14)	0.52372 (17)	0.40425 (13)	0.0233 (4)	
H17A	0.7512	0.4876	0.4600	0.035*	
H17B	0.6750	0.6105	0.4188	0.035*	
H17C	0.7098	0.5242	0.3492	0.035*	

### Atomic displacement parameters (Å^2^)

**Table:**

	*U*^11^	*U*^22^	*U*^33^	*U*^12^	*U*^13^	*U*^23^
Ni1	0.00850 (13)	0.00775 (13)	0.00768 (13)	0.00027 (9)	0.00159 (10)	0.00033 (9)
Cl1	0.0248 (2)	0.0307 (2)	0.0230 (2)	−0.00606 (17)	0.00789 (18)	−0.00259 (17)
O1	0.0143 (5)	0.0135 (5)	0.0110 (5)	0.0022 (4)	0.0055 (4)	0.0018 (4)
O2	0.0457 (8)	0.0154 (6)	0.0323 (7)	0.0119 (5)	0.0293 (6)	0.0083 (5)
O3	0.0180 (5)	0.0122 (5)	0.0103 (5)	0.0014 (4)	0.0008 (4)	−0.0013 (4)
O4	0.0136 (5)	0.0093 (5)	0.0121 (5)	0.0011 (4)	0.0034 (4)	0.0006 (4)
N1	0.0114 (6)	0.0109 (6)	0.0102 (6)	−0.0004 (5)	0.0027 (5)	0.0001 (5)
N2	0.0143 (6)	0.0175 (7)	0.0098 (6)	0.0038 (5)	0.0009 (5)	0.0004 (5)
C1	0.0158 (7)	0.0125 (7)	0.0126 (7)	−0.0021 (6)	0.0061 (6)	−0.0011 (6)
C2	0.0181 (7)	0.0132 (7)	0.0166 (7)	0.0037 (6)	0.0106 (6)	0.0016 (6)
C3	0.0185 (8)	0.0232 (9)	0.0194 (8)	0.0028 (6)	0.0081 (7)	0.0026 (7)
C4	0.0288 (9)	0.0322 (10)	0.0175 (8)	0.0105 (8)	0.0104 (7)	0.0082 (7)
C5	0.0380 (10)	0.0253 (9)	0.0269 (9)	0.0072 (8)	0.0228 (9)	0.0114 (7)
C6	0.0293 (9)	0.0199 (9)	0.0286 (9)	−0.0026 (7)	0.0193 (8)	0.0018 (7)
C7	0.0212 (8)	0.0181 (8)	0.0175 (8)	0.0008 (6)	0.0098 (7)	0.0002 (6)
C8	0.0125 (7)	0.0120 (7)	0.0131 (7)	0.0019 (6)	0.0018 (6)	0.0028 (6)
C9	0.0169 (7)	0.0093 (7)	0.0164 (7)	−0.0002 (6)	0.0039 (6)	0.0010 (6)
C10	0.0137 (7)	0.0125 (7)	0.0133 (7)	−0.0022 (6)	0.0033 (6)	−0.0019 (6)
C11	0.0117 (7)	0.0120 (7)	0.0097 (7)	0.0005 (5)	0.0022 (6)	−0.0006 (5)
C12	0.0144 (7)	0.0097 (7)	0.0113 (7)	0.0006 (5)	0.0032 (6)	0.0012 (5)
C13	0.0114 (7)	0.0089 (7)	0.0118 (7)	−0.0023 (5)	0.0009 (6)	−0.0005 (5)
C14	0.0223 (9)	0.0383 (10)	0.0129 (8)	0.0119 (7)	0.0042 (7)	−0.0016 (7)
C15	0.0259 (10)	0.0442 (12)	0.0291 (10)	0.0087 (8)	−0.0003 (8)	−0.0213 (9)
C16	0.0159 (7)	0.0144 (8)	0.0147 (7)	0.0054 (6)	0.0012 (6)	0.0021 (6)
C17	0.0169 (8)	0.0271 (9)	0.0214 (9)	0.0006 (7)	0.0033 (7)	−0.0046 (7)

### Geometric parameters (Å, °)

**Table:**

Ni1—O1^i^	2.0336 (10)	C7—C2	1.390 (2)
Ni1—O1	2.0336 (10)	C7—C6	1.386 (2)
Ni1—O4	2.0867 (10)	C8—C9	1.387 (2)
Ni1—O4^i^	2.0867 (10)	C8—H8	0.93
Ni1—N1^i^	2.1181 (12)	C9—H9	0.93
Ni1—N1	2.1181 (12)	C10—C9	1.379 (2)
Cl1—C7	1.7466 (17)	C10—H10	0.93
O1—C1	1.2616 (17)	C11—C10	1.392 (2)
O2—C1	1.2435 (18)	C11—C12	1.384 (2)
O3—C13	1.2429 (17)	C12—H12	0.93
O4—H41	0.820 (15)	C13—N2	1.3369 (18)
O4—H42	0.854 (15)	C13—C11	1.501 (2)
N1—C8	1.3405 (19)	C14—H14A	0.97
N1—C12	1.3452 (18)	C14—H14B	0.97
N2—C14	1.470 (2)	C15—C14	1.522 (3)
N2—C16	1.4728 (19)	C15—H15A	0.96
C2—C3	1.392 (2)	C15—H15B	0.96
C2—C1	1.510 (2)	C15—H15C	0.96
C3—C4	1.394 (2)	C16—H16A	0.97
C3—H3	0.93	C16—H16B	0.97
C4—H4	0.93	C17—C16	1.519 (2)
C5—C4	1.382 (3)	C17—H17A	0.96
C5—H5	0.93	C17—H17B	0.96
C6—C5	1.380 (2)	C17—H17C	0.96
C6—H6	0.93		
			
O1^i^—Ni1—O1	180.0	C6—C7—C2	121.94 (15)
O1^i^—Ni1—O4	93.00 (4)	C6—C7—Cl1	119.13 (13)
O1—Ni1—O4	87.00 (4)	N1—C8—C9	122.93 (14)
O1^i^—Ni1—O4^i^	87.00 (4)	N1—C8—H8	118.5
O1—Ni1—O4^i^	93.00 (4)	C9—C8—H8	118.5
O1^i^—Ni1—N1^i^	90.70 (4)	C8—C9—H9	120.3
O1—Ni1—N1^i^	89.30 (4)	C10—C9—C8	119.41 (14)
O1^i^—Ni1—N1	89.30 (4)	C10—C9—H9	120.3
O1—Ni1—N1	90.70 (4)	C9—C10—C11	118.10 (13)
O4—Ni1—O4^i^	180.0	C9—C10—H10	121.0
O4—Ni1—N1^i^	92.55 (4)	C11—C10—H10	121.0
O4^i^—Ni1—N1^i^	87.45 (4)	C10—C11—C13	118.90 (13)
O4—Ni1—N1	87.45 (4)	C12—C11—C10	119.09 (13)
O4^i^—Ni1—N1	92.55 (4)	C12—C11—C13	121.91 (13)
N1^i^—Ni1—N1	180.00 (7)	N1—C12—C11	122.94 (13)
C1—O1—Ni1	127.35 (9)	N1—C12—H12	118.5
Ni1—O4—H41	104.4 (15)	C11—C12—H12	118.5
Ni1—O4—H42	125.8 (15)	O3—C13—N2	122.60 (13)
H41—O4—H42	109 (2)	O3—C13—C11	118.39 (13)
C8—N1—Ni1	122.58 (10)	N2—C13—C11	119.01 (13)
C8—N1—C12	117.45 (12)	N2—C14—C15	112.80 (15)
C12—N1—Ni1	119.97 (9)	N2—C14—H14A	109.0
C13—N2—C14	124.98 (13)	N2—C14—H14B	109.0
C13—N2—C16	118.44 (12)	C15—C14—H14A	109.0
C14—N2—C16	116.10 (12)	C15—C14—H14B	109.0
O1—C1—C2	114.14 (12)	H14A—C14—H14B	107.8
O2—C1—O1	127.09 (14)	C14—C15—H15A	109.5
O2—C1—C2	118.77 (13)	C14—C15—H15B	109.5
C3—C2—C1	121.41 (14)	C14—C15—H15C	109.5
C7—C2—C1	120.65 (14)	H15A—C15—H15B	109.5
C7—C2—C3	117.87 (14)	H15A—C15—H15C	109.5
C2—C3—C4	120.80 (16)	H15B—C15—H15C	109.5
C2—C3—H3	119.6	N2—C16—C17	111.60 (13)
C4—C3—H3	119.6	N2—C16—H16A	109.3
C3—C4—H4	120.1	N2—C16—H16B	109.3
C5—C4—C3	119.74 (16)	C17—C16—H16A	109.3
C5—C4—H4	120.1	C17—C16—H16B	109.3
C6—C5—C4	120.52 (15)	H16A—C16—H16B	108.0
C6—C5—H5	119.7	C16—C17—H17A	109.5
C4—C5—H5	119.7	C16—C17—H17B	109.5
C5—C6—C7	119.08 (16)	C16—C17—H17C	109.5
C5—C6—H6	120.5	H17A—C17—H17B	109.5
C7—C6—H6	120.5	H17A—C17—H17C	109.5
C2—C7—Cl1	118.94 (12)	H17B—C17—H17C	109.5
			
O4—Ni1—O1—C1	172.55 (12)	C1—C2—C3—C4	−174.81 (14)
O4^i^—Ni1—O1—C1	−7.45 (12)	C7—C2—C3—C4	2.0 (2)
N1^i^—Ni1—O1—C1	−94.85 (12)	C2—C3—C4—C5	−0.2 (2)
N1—Ni1—O1—C1	85.15 (12)	C6—C5—C4—C3	−1.3 (3)
O1^i^—Ni1—N1—C8	58.61 (11)	C7—C6—C5—C4	0.8 (3)
O1—Ni1—N1—C8	−121.39 (11)	Cl1—C7—C2—C1	−5.36 (19)
O1^i^—Ni1—N1—C12	−121.15 (11)	Cl1—C7—C2—C3	177.78 (12)
O1—Ni1—N1—C12	58.85 (11)	C6—C7—C2—C1	174.35 (14)
O4—Ni1—N1—C8	151.65 (11)	C6—C7—C2—C3	−2.5 (2)
O4^i^—Ni1—N1—C8	−28.35 (11)	Cl1—C7—C6—C5	−179.16 (12)
O4—Ni1—N1—C12	−28.11 (11)	C2—C7—C6—C5	1.1 (2)
O4^i^—Ni1—N1—C12	151.89 (11)	N1—C8—C9—C10	1.6 (2)
Ni1—O1—C1—O2	−9.8 (2)	C11—C10—C9—C8	1.1 (2)
Ni1—O1—C1—C2	170.38 (9)	C12—C11—C10—C9	−2.7 (2)
Ni1—N1—C8—C9	177.78 (11)	C13—C11—C10—C9	−179.11 (13)
C12—N1—C8—C9	−2.5 (2)	C10—C11—C12—N1	1.8 (2)
Ni1—N1—C12—C11	−179.49 (11)	C13—C11—C12—N1	178.16 (13)
C8—N1—C12—C11	0.7 (2)	O3—C13—N2—C14	−174.33 (15)
C13—N2—C14—C15	−110.16 (17)	O3—C13—N2—C16	−2.5 (2)
C16—N2—C14—C15	77.88 (18)	C11—C13—N2—C14	5.4 (2)
C13—N2—C16—C17	−90.03 (17)	C11—C13—N2—C16	177.22 (12)
C14—N2—C16—C17	82.48 (17)	O3—C13—C11—C10	61.59 (19)
C3—C2—C1—O1	85.51 (18)	O3—C13—C11—C12	−114.75 (16)
C3—C2—C1—O2	−94.30 (19)	N2—C13—C11—C10	−118.19 (15)
C7—C2—C1—O1	−91.23 (17)	N2—C13—C11—C12	65.48 (19)
C7—C2—C1—O2	88.96 (19)		

Symmetry codes: (i) −*x*, −*y*+1, −*z*.

### Hydrogen-bond geometry (Å, °)

**Table:**

*D*—H···*A*	*D*—H	H···*A*	*D*···*A*	*D*—H···*A*
O4—H41···O2^i^	0.82 (2)	1.86 (2)	2.6267 (17)	155 (2)
O4—H42···O3^ii^	0.85 (2)	1.93 (2)	2.7826 (15)	172 (2)

Symmetry codes: (i) −*x*, −*y*+1, −*z*; (ii) −*x*+1/2, *y*−1/2, −*z*+1/2.
